# Supporting access to alcohol and other drug treatment: the Los Angeles County screening, brief intervention, and referral to treatment project for short-term jail detainees

**DOI:** 10.1186/1940-0640-7-S1-A57

**Published:** 2012-10-09

**Authors:** Anne Lee, Richard Rawson, Rebecca Beattie

**Affiliations:** 1 Neuropsychiatric Institute, University of California at Los Angeles, Los Angeles, CA, USA; 2 Department of Psychiatry, University of California at Los Angeles, Los Angeles, CA, USA; 3 University of California at Los Angeles Integrated Substance Abuse Programs, Los Angeles, CA, USA

## 

The University of California at Los Angeles Integrated Substance Abuse Program (UCLA ISAP) was contracted to evaluate the Los Angeles County screening, brief intervention, and referral to treatment project (LA SBIRT). This project was funded by the US Substance Abuse and Mental Health Services Administration to provided SBIRT for short-term-stay detainees. Elements of the program include increasing access to care and reducing alcohol and other drug (AOD) use by screening with the Alcohol, Smoking, and Substance Use Involvement Screening Test (ASSIST), which assesses risk of developing problems related to AOD use; performing a motivational brief intervention based on the results of the ASSIST; and, when indicated, referring detainees to a course of brief treatment sessions. The university’s ISAP used the Government Performance and Results Act tool (GPRA) and ASSIST data to describe the population and assess outcomes. They also conducted a process evaluation. According to results, risky alcohol, cannabis, and tobacco use were high in this group. Among all participants who completed the ASSIST, 95% scored either high or moderate risk for at least one substance. Fewer participants reported using all AODs at follow-up than at baseline, and those who used substances reported using all AODs on fewer days at follow-up. Participants also had less involvement with the criminal justice system at follow-up and received more outpatient services. This SBIRT project was a successful collaboration between the greater Los Angeles criminal justice system and substance abuse treatment networks and offered access to treatment for thousands of detainees. A key lesson learned was that conducting the ASSIST in the jail setting decrease dropout from treatment in this transient population. Locating clients for follow-up was extremely difficult, thus, incentives plus tracking and locating are crucial. Staff felt the ASSIST and motivational interviewing was extremely well-suited to this population. The success of this project depends upon ongoing training, supervision, and collaboration among the stakeholders.

